# Processing of Emotional Faces in Patients with Chronic Pain Disorder: An Eye-Tracking Study

**DOI:** 10.3389/fpsyt.2018.00063

**Published:** 2018-03-05

**Authors:** Katrin Elisabeth Giel, Sarah Paganini, Irena Schank, Paul Enck, Stephan Zipfel, Florian Junne

**Affiliations:** ^1^Department of Psychosomatic Medicine and Psychotherapy, Medical University Hospital Tübingen, Tübingen, Germany; ^2^Department of Rehabilitation Psychology and Psychotherapy, Institute of Psychology, Albert Ludwigs University of Freiburg, Freiburg, Germany

**Keywords:** attention, chronic pain, depression, emotion, face, eye tracking

## Abstract

**Objective:**

Problems in emotion processing potentially contribute to the development and maintenance of chronic pain. Theories focusing on attentional processing have suggested that dysfunctional attention deployment toward emotional information, i.e., attentional biases for negative emotions, might entail one potential developmental and/or maintenance factor of chronic pain.

**Methods:**

We assessed self-reported alexithymia, attentional orienting to and maintenance on emotional stimuli using eye tracking in 17 patients with chronic pain disorder (CP) and two age- and sex-matched control groups, 17 healthy individuals (HC) and 17 individuals who were matched to CP according to depressive symptoms (DC). In a choice viewing paradigm, a dot indicated the position of the emotional picture in the next trial to allow for strategic attention deployment. Picture pairs consisted of a happy or sad facial expression and a neutral facial expression of the same individual. Participants were asked to explore picture pairs freely.

**Results:**

CP and DC groups reported higher alexithymia than the HC group. HC showed a previously reported *emotionality bias* by preferentially orienting to the emotional face and preferentially maintaining on the happy face. CP and DC participants showed no facilitated early attention to sad facial expressions, and DC participants showed no facilitated early attention to happy facial expressions, while CP and DC participants did. We found no group differences in attentional maintenance.

**Conclusion:**

Our findings are in line with the clinical large overlap between pain and depression. The blunted initial reaction to sadness could be interpreted as a failure of the attentional system to attend to evolutionary salient emotional stimuli or as an attempt to suppress negative emotions. These difficulties in emotion processing might contribute to etiology or maintenance of chronic pain and depression.

## Introduction

Explanatory models of chronic pain have included specific emotional states and problems in emotion processing as factors potentially contributing to the development and maintenance of chronic pain (1, 2). Neurobiological findings show that brain circuits processing pain are modulated by emotions (1, 2). For example, a recent review concludes that pain catastrophizing—which is defined as difficulty shifting attention away from painful or threatening stimuli—is associated with changes in gray matter morphology and resting state functional connectivity in brain areas involved in pain, motor, somatosensory, and affective–cognitive processing (3). Not only difficulties in identifying, describing, and expressing one’s own emotions (i.e., alexithymia) but also the experience of intensive negative emotions might trigger and modulate pain (1, 4–6). These difficulties might also become evident in attentional processing of emotional information and could comprise *attentional biases*, including facilitated orienting to salient stimuli in early processing stages and/or attentional maintenance in later processing stages (7, 8). Attentional biases give insight into mechanisms of information processing and, at the same time, can represent important maintenance factors of mental disorders. For instance, an attentional bias for pain-related information has been reported in patients with chronic pain (9–11), although a few constrictions have to be taken into account: such a bias can also be found in healthy volunteers, it seems to depend on the type of pain-related information and exposure time and was, e.g., stronger for words that reflect the sensory characteristics of pain and for supraliminal presentation (10).

Emotional stimuli catch attention very early and are preferentially processed (12). The human attentional system differentiates emotional content from neutral content fast and robust in an early processing stage (13). This prioritization probably reflects the evolutionary importance of emotional information, for instance, the identification of own and others’ emotional expressions are important to shape successful social interactions. In line with this, there is evidence for an attentional bias for emotional faces in healthy participants (14) and for altered attention deployment to emotional information in affective disorders. Depressed individuals show attentional biases for dysphoric emotional stimuli, including preferred early orienting and maintenance on them, while maintenance on positive stimuli is reduced (15, 16); however, it has to be considered that this phenomenon seems to be moderated by age (17, 18). Depression is often a comorbidity in chronic pain (2), while the precise (causal) relationship of both conditions remains unclear and we hence also use the descriptive term “overlap” to indicate this. One shared phenomenon could be an insensitivity to reward (2, 16), leading to an “anhedonia bias,” i.e., a failure of the attentional system to attend to rewarding (emotional) stimuli (1, 16).

Dysfunctional processing of positive and negative emotional information might also play a role in development and maintenance of chronic pain. Few studies have investigated attentional processing of emotional content in chronic pain (19–21); a recent study reports alterations of emotional and attentional information processing in patients with fibromyalgia (21), and another study found an attentional bias for negative emotional words in fibromyalgia patients based on the Stroop task (20). Rossello et al. (21) presented video tours through an external environment under induced pleasant, unpleasant, or neutral affective state and assessed different psychophysiological measures in response to these environments. Patients with fibromyalgia showed abnormal brain functioning and autonomic cardiovascular control during affective processing, but this was not specific for the unpleasant affective state. In the study by Duschek et al. (20), participants were asked to name the color of an adjective that was negative, positive, or neutral. Patients with fibromyalgia needed longer response times in the case of negative adjectives that were interpreted as an attentional bias for negative information. Recently, to allow insights into the time course of attention deployment, several researchers in the field have suggested the use of eye-tracking methodology (7, 22). As compared to other experimental methods, eye-tracking allows for the assessment of overt visual attention deployment with a very high time and space resolution, which is especially of value when investigating attentional biases as they occur both in early and later processing stages, and they might be based on relatively small time differences. To date, only few studies have used eye tracking in clinical populations with chronic pain. A recent study investigated the processing of injury-related vs. neutral pictures in patients with chronic pain, and healthy volunteers found that patients might be characterized by general avoidance of exploring both picture categories as well as a late maintenance bias for injury-related pictures (23). We used eye-tracking methodology to investigate attentional orienting and maintenance to standardized emotional facial expressions. In a choice viewing paradigm, we presented picture pairs consisting of an emotional picture, depicting a facial expression of either happiness or sadness, while the control picture depicted a neutral facial expression. A cue indicated the position of the emotional picture. We used this cue as it allows for strategic attention deployment, that is, based on this prior information, participants can decide to avoid or to explore the emotional picture. We considered this early strategic processing of emotional content especially interesting in chronic pain patients given potential problems dealing with negative or threatening information in terms of pain catastrophizing as outlined above. We investigated patients with chronic pain disorder (CP), healthy controls (HC), and a control group of individuals who were matched to CP according to level of depressive symptoms (DC). Our specific interest was to investigate if potential alterations in emotion processing are specific for chronic pain or might be influenced by depression.

Regarding alexithymia, we expected to be able to replicate earlier evidence which found increased difficulties in identifying, describing, and expressing one’s own emotions in patients with chronic pain (6). Therefore, we hypothesize that CP reported more problems recognizing and describing own emotions as compared to the healthy control group. With respect to attention deployment, a prioritization of emotional information by the attentional system has been shown (13). Based on this, we expected to replicate that HC showed an early and maintained attentional bias for emotional faces. The anhedonia bias hypothesis suggests that patients with depression and patients with chronic pain might be less sensitive to reward, which entails that their attentional system might not adequately respond to rewarding (emotional) stimuli (1, 16). Moreover, earlier findings in patients with chronic pain from a different paradigm showed an attentional bias for negative information (20) and evidence from depression research has demonstrated that adults with depression show attentional biases for dysphoric emotional stimuli, including preferred early orienting and maintenance on them, while maintenance on positive stimuli is reduced (15, 16). Therefore, we expected that CP and DC showed facilitated orienting to and longer maintenance on negative emotions (sad faces), while less orienting to and maintaining on positive emotions (happy faces).

## Materials and Methods

### Participants

We assessed three groups of participants: (1) an experimental group of CP, (2) a control group of healthy individuals (HC), and (3) a control group of individuals with depressive symptoms (DC) but without pain.

We recruited 17 female and male CP according to DSM-IV (24) from the inpatient, day patient, and outpatient programs of the Department of Psychosomatic Medicine and Psychotherapy at the Medical University Hospital Tübingen and *via* announcements from the general population. In order to be included, patients had to report pain for at least 6 months and to report current pain intensity in the last 4 weeks of at least 4 on a visual analogue scale (VAS). According to the classification of pain proposed for ICD-11 (25), seven patients reported primary pain, seven patients suffered from musculoskeletal pain, two from headache, and one patient reported visceral pain. 40% of CP fulfilled criteria of at least one other mental disorder, mostly mood and anxiety disorders, according to DSM-IV.

To each of the CP, we selected two control participants who were matched according to age and sex, and one of these participants was additionally matched according to severity of depression symptoms (clinical control group DC). Participants of the HC and DC groups were predominantly recruited from the general population; some participants of the DC group were also recruited from clinical services of the Department of Psychosomatic Medicine and Psychotherapy at the Medical University Hospital Tübingen.

This results in a total sample of *N* = 51. The sex ratio slightly differs between groups for experimental data as single participants had to be excluded from data analysis (see below).

Exclusion criteria for all participants comprise impaired and non-corrected vision, psychosis, bipolar I disorder or substance addiction according to DSM-IV, heavy nicotine use, and psychotropic medication except for selective serotonin reuptake inhibitors in a stable dosage.

Participants of both control groups were free of clinically relevant pain. Participants of the healthy control group had no history of any mental or serious somatic disorder.

### Stimuli

We used standardized stimulus material derived from the Karolinska Directed Emotional Faces (KDEF) database (26). The KDEF is a set of totally 4,900 pictures of human facial expressions of emotion. The set contains faces of 70 amateur actors (35 females and 35 males), each displaying 7 different emotional expressions, each expression being photographed (twice) from 5 different angles. The displayed emotions comprise neutral, happy, angry, afraid, disgusted, sad, and surprised. For the presents study, we selected picture pairs of a happy vs. neutral expression and sad vs. neutral expression (by the same actor, respectively). Pictures of different actors were selected for the *happy* condition and the *sad* condition. We used only frontal pictures. The picture set consisted of 26 picture pairs in the *happy* condition, displaying 13 female and 13 male faces, and 25 picture pairs in the *sad* condition, displaying 13 female and 12 male faces.

The individual pictures from each pair were presented in two opposing corners of the computer screen (top left/bottom right or top right/bottom left) against a gray background. The target and control stimuli locations were balanced across trials. All pictures covered 377 × 281 pixel.

### Experimental Paradigm

**Figure 1:**
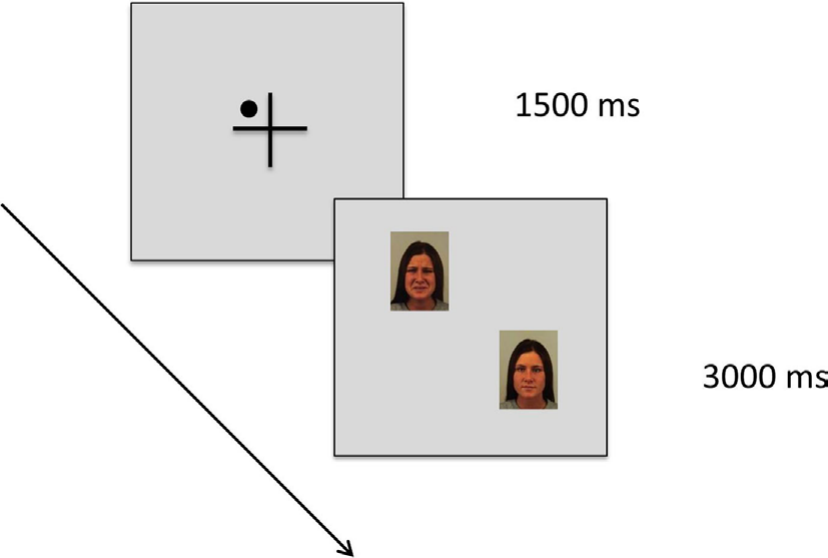
Schematic diagram of the cued choice viewing paradigm. Each trial starts with a fixation cross. Within this cross, a small dot was presented in one of the four quadrants as a cue, and this cue indicated with 100% reliability the position of the emotional picture in the next trial (in this example: the face with a sad expression). Then, a picture pair was presented, consisting of an emotional picture paired with a neutral picture (in this example: Karolinska Directed Emotional Faces image ID AF30NES and AF30SAS). We used a free viewing task, that is, participants were asked to explore the picture pairs freely as if they were watching television. There was no instruction associated with the cue.

We used a choice viewing paradigm (27), which has previously been used by our own group and others as a paradigm sensitive to individual differences in hedonic response and emotional behavior (Figure 1) (27–30).

In the present choice viewing paradigm, we presented picture pairs for 3 s each, consisting of an emotional picture paired with a neutral picture. The emotional picture depicted a facial expression of either happiness or sadness, while the control picture depicted a neutral facial expression of the same individual (see description of stimulus material above). Each picture pair was preceded by a central fixation cross presented for 1,500 ms. Participants were asked to fixate this cross. Within this cross, a small dot was presented in one of the four quadrants (top left/bottom right or top right/bottom left) as a cue, and this cue indicated with 100% reliability the position of the emotional picture in the next trial. Participants were told about this relationship, but there was no other instruction associated with this cue. We used a free viewing task, asking participants to explore the picture pairs freely as if they were watching television.

Our cued choice viewing paradigm thereby targets the goal directed attentional system as it allows for strategic attention deployment, especially in early processing stages. Gaze behavior in this paradigm provides information about how patients with chronic pain deal with the anticipated confrontation with positive vs. negative emotional information.

The emotion expressed by the facial stimuli was blocked, and a sequence of blocks (*happy* vs. *sad*) were randomized.

### Procedure

The study was conducted on two separate appointments: a first appointment for diagnostic purposes and a second appointment comprising the experimental task.

During the diagnostic assessment, we conducted the structured clinical interview for DSM-IV (SCID-I) (31) and assessed basic demographic characteristics. Onset, duration, localization, quality, and intensity of pain in CP were assessed using items of the German Pain Questionnaire. We additionally assessed strongest intensity of pain during the last 4 weeks using a VAS ranging from 0 (no pain) to 10 (strongest conceivable pain). All participants completed validated self-report instruments assessing depressive symptoms [Beck Depression Inventory (BDI)] (32) and general psychopathology (Patient Health Questionnaire) (33).

In order to assess difficulties recognizing and describing own emotions, we used the Toronto Alexithymia Scale 26 (TAS26) (34). The TAS is a self-report instrument very commonly used to assess alexithymia as a multidimensional concept, consisting of three subscales: difficulty identifying feelings, difficulty describing feelings, and externally oriented thinking.

For the experimental session, participants arrived at the laboratory during an afternoon appointment to control for circadian variation in the sample. Participants were asked to refrain from smoking and caffeine 3 h before testing. The eye-tracking system was adjusted to each individual and we performed a 13-point-calibration before starting the eye-tracking task. After completion of the task, participants rated all stimuli for pleasantness using a VAS ranging from −4 (maximum unpleasant) to +4 (maximum pleasant). Finally, we assessed current pain intensity using again a VAS ranging from 0 to 10 and emotional burden with a VAS ranging from 0 (no burden) to 10 (strongest conceivable burden).

### Apparatus

Data were assessed using the remote Eye Tracking System iView X Hi-Speed (SensoMotoric Instruments GmbH, Berlin, Germany) with a sampling rate of 500 Hz.

### Ethical Consideration

The study was approved by the Ethics Committee of the Medical Faculty and the University Hospital Tübingen at the Eberhard Karls University Tübingen. This study was carried out in accordance with the recommendations of the Ethics Committee of the Medical Faculty and the University Hospital Tübingen at the Eberhard Karls University Tübingen with written informed consent from all subjects. All subjects gave written informed consent in accordance with the Declaration of Helsinki.

### Data Preparation and Analyses

Raw gaze data were analyzed using BeGaze 2.0 (SensoMotoric Instruments, Berlin, Germany).

We excluded trials from data analysis where participants had not fixated on the fixation cross or data with insufficient data quality. We excluded participants with less than 50% of trials analyzable. This resulted in the exclusion of six participants from the block with happy facial expressions and of three participants from the block with sad facial expressions.

We defined areas of interests (AOIs) which comprised the face on each of the depicted photographs and analyzed gaze data exclusively for these AOIs.

We defined the following two gaze measures which were previously used in eye-tracking research [e.g., Ref. (22, 29)]: (1) the position of the initial fixation after the trial’s onset as a measure of attentional orienting and (2) percentage of total gaze duration on the AOI during a trial as a measure of continuous attentional engagement or maintenance. In order to perform exploratory correlation analyses, we calculated attentional bias scores: (1) a gaze direction bias score by subtracting the percentage of initial fixations on neutral stimuli from the percentage of initial fixations on emotional stimuli and (2) a gaze duration bias score by subtracting the total gaze duration on neutral stimuli from the total gaze duration on emotional stimuli. Positive scores reflect a bias toward emotional stimuli.

Data were analyzed using SPSS version 21.0 (IBM). All tests were two-tailed. We chose an α-level of 0.05 as threshold of statistical significance.

Based on the reported results of one primary outcome measure (i.e., initial orienting to negative emotion), a *post hoc* power analysis was performed, indicating that the group sizes of *n* = 17 obtained 76.3% power to detect differences between the CP and the HC group in this variable using a two-tailed statistical significance criterion of 5%.

To analyze potential baseline group differences in demographic and clinical variables, we used univariate analysis of variance (ANOVA) with *group* as between subject factor. Fisher’s least significant difference was used to identify *post hoc* group differences. To analyze gaze data, we used repeated measure ANOVAs with stimulus type (emotional facial expression vs. neutral facial expression) as within subject factor and group (CP, DC, and HC) as between subject factor. We performed Scheffé tests to identify *post hoc* group differences as this test procedure is considered a more conservative *post hoc* analysis. In order to assess within group biases, we used one-sample *t*-tests. We report effect sizes for group differences using η^2^, with η^2^ < 0.06 representing a small effect, 0.06 < η^2^ < 0.14 representing a medium effect, and η^2^ ≥ 0.14 representing a large effect.

We performed exploratory correlational analysis using Pearson’s correlations, assessing potential relationships between attention deployment (i.e., the gaze direction bias score and the gaze duration bias score) on the one hand and self-report continuous measures of alexithymia (TAS26 total score), depression (BDI score), and pain (current pain intensity and strongest pain intensity in the last 4 weeks).

## Results

### Participants

Table 1 gives and overview on demographic and clinical characteristics of the study sample. The matching was successful: the three groups are comparable with respect to age, and the group CP and DC are comparable with respect to depressive symptoms. Level of depressive symptoms in CP and DC participants was classified as “mild” according to BDI classification.

**Table 1:** Demographic and clinical baseline characteristics of the study groups.

Group	CP (*n* = 17)	DC (*n* = 17)	HC (*n* = 17)	Group differences	*p*-Value
Females (*n*)	7	5	6		

	**M ± SD**	**M ± SD**	**M ± SD**		

Age (years)	39.8 ± 13.7	38.2 ± 12.3	39.2 ± 13.7	*F* (2, 48) = 0.060	0.942
Onset of pain (years)	4.9 ± 5.5	N/A	N/A		
Current pain intensity^c^	4.7 ± 2.0*	0.8 ± 1.4	0.1 ± 0.3	*F* (2, 47) = 50.254	<0.001
Strongest pain intensity in the last 4 weeks^c^	7.7 ± 1.8*	3.1 ± 2.7	2.7 ± 2.0	*F* (2, 47) = 27.979	<0.001
Depressive symptoms^a^	13.1 ± 8.8	13.8 ± 8.5	1.4 ± 1.5*	*F* (2, 48) = 16.227	<0.001
Somatic symptoms^b^	10.5 ± 4.5*	5.0 ± 3.2	3.2 ± 2.3	*F* (2, 47) = 19.765	<0.001
Current emotional burden^c^	4.6 ± 2.6	4.4 ± 2.7	1.7 ± 2.3*	*F* (2, 47) = 7.161	0.002
Alexithymia^d^	2.5 ± 0.6	2.4 ± 0.6	2.0 ± 0.4*	*F* (2, 45) = 3.224	0.049

*CP, patients with a chronic pain disorder; DC, control participants with depressive symptoms; HC, healthy control participants*.

*^a^Assessed using the German version of the Beck Depression Inventory*.

*^b^Assessed using the German version of the Patient Health Questionnaire*.

*^c^Assessed using a visual analogue scale ranging from 0 to 10*.

*^d^Assessed using the German version of the Toronto Alexithymia Scale 26 (*n* = 16 for HC and *n* = 15 for DC)*.

*Indicates the group that significantly differs from the other groups.

### Alexithymia

Patients with chronic pain disorder and DC patients showed significantly higher scores on the TAS26 than HC (Table 1), indicating that both groups have more overall difficulties identifying and communicating own feelings.

### Valence Rating

**Figure 2:**
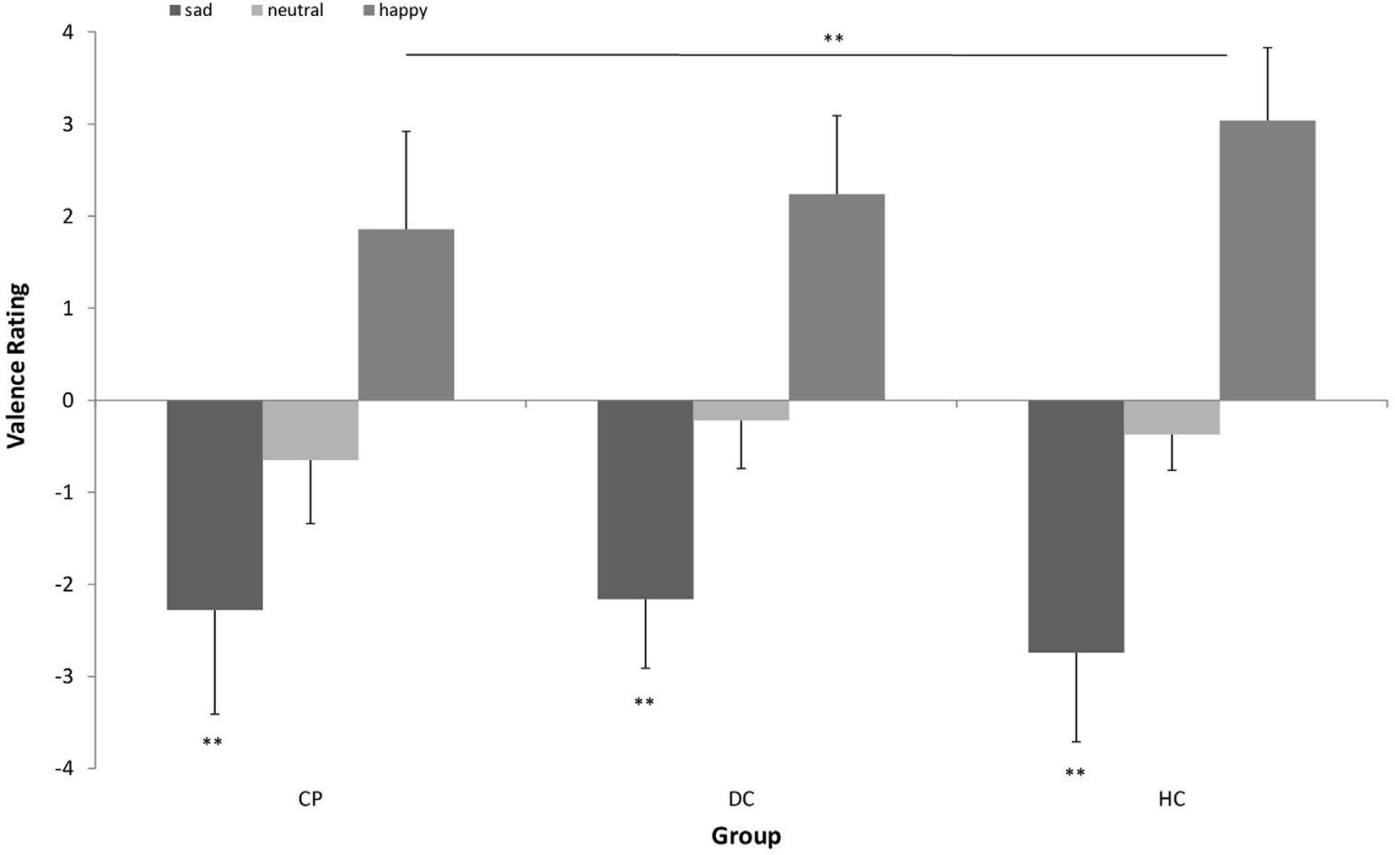
Valence rating of positive (happy facial expression) vs. negative (sad facial expression) emotional pictures vs. neutral pictures (neutral facial expression) in *n* = 17 patients with chronic pain disorder (CP), *n* = 17 individuals with depressive symptoms (DC), and *n* = 17 healthy individuals (HC). Sad faces were rated as significantly less pleasant than neutral and happy faces by all groups. The CP group rated the happy faces as significantly less pleasant than the HC group (**p < 0.01).

Figure 2 shows results of the valence rating. We found a main effect of stimulus type [*F* (2) = 348.709; *p* < 0.001; η^2^ = 0.879] with a large effect size. Sad faces were rated as significantly less pleasant than neutral and happy faces. We also found a significant stimulus × group interaction [*F* (4) = 4.078; *p* = 0.015; η^2^ = 0.145] with a large effect size. Participants were not different with respect to their valence rating of sad and neutral faces [*F* (2, 48) = 1.963; *p* = 0.152], however, there was a significant effect for happy faces [*F* (2, 48) = 6.654; *p* = 0.003]. *Post hoc*-analyses revealed that the CP group rated the happy faces as significantly less pleasant than the HC group (*p* = 0.004).

### Attentional Orienting to Emotional Stimuli

**Figure 3:**
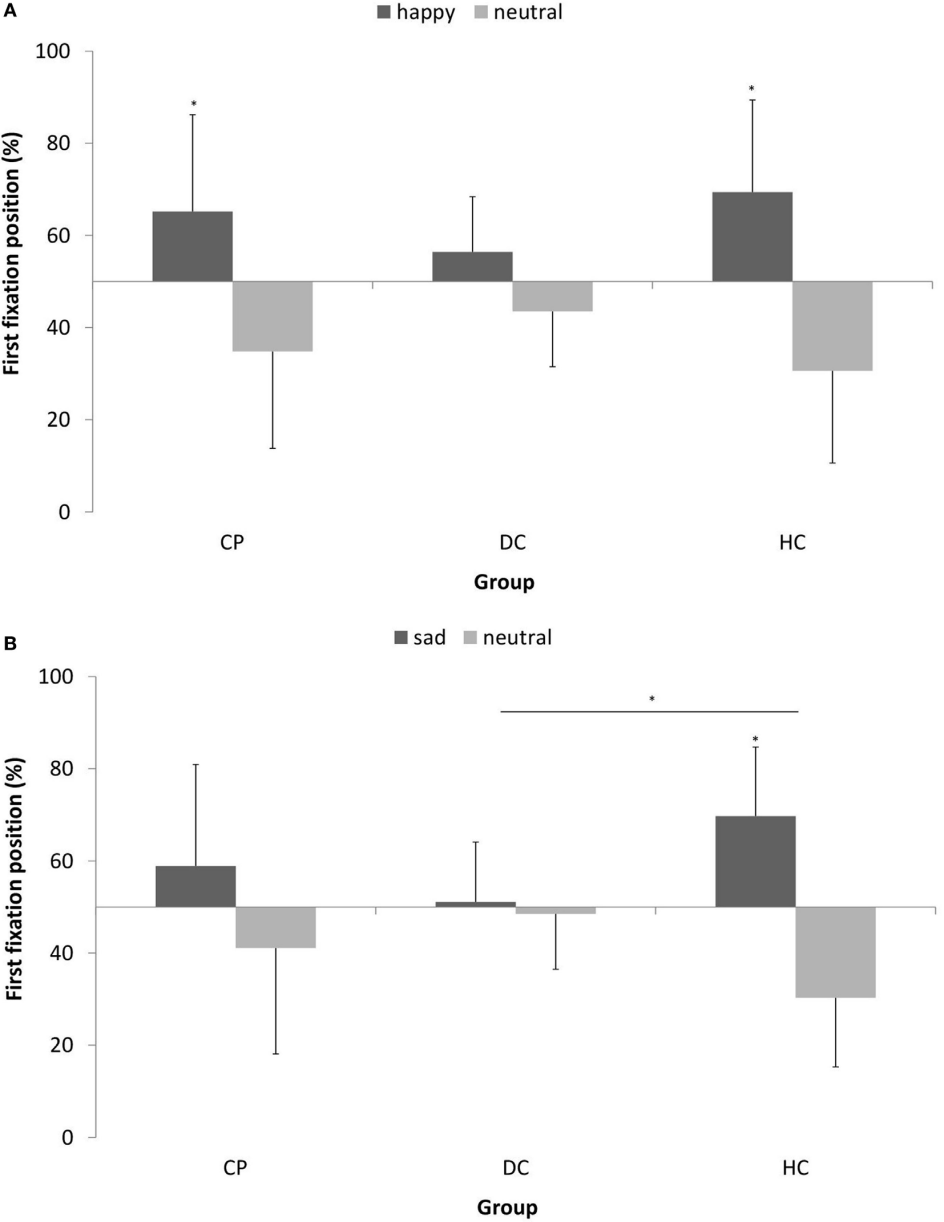
Mean (±SD) percentage of first fixation position on **(A)** happy vs. neutral facial expression in *n* = 15 patients with chronic pain disorder (CP), *n* = 15 individuals with depressive symptoms (DC), and *n* = 15 healthy individuals (HC) and **(B)** sad vs. neutral facial expression in *n* = 16 CP, *n* = 16 individuals with depressive symptoms (DC), and *n* = 16 healthy individuals (HC). The CP group and HC group oriented their attention significantly more often first to happy faces, while the DC group showed no preference for happy faces in attentional orienting (*p < 0.05).

Figure 3 shows the pattern of attentional orienting to emotional stimuli.

With respect to *happy* vs. neutral faces, we found a main effect of stimulus type [*F* (1, 42) = 21.604; *p* < 0.001; η^2^ = 0.34] with a large effect size, which means, there was a general bias in attentional orienting toward happy faces as compared to neutral faces. When looking at single participant groups, this tendency to orient attention first to happy faces was significant in the CP group [*t*(14) = 2.380; *p* = 0.032] and in the HC group [*t*(14) = 3.868; *p* = 0.002], while the DC group showed no preference for happy faces in attentional orienting [*t*(14) = 1.885; *p* > 0.05]. We found no interaction effect.

With respect to *sad* vs. neutral faces, we found no main effect of stimulus type, but a significant stimulus × group interaction [*F* (2, 45) = 3.611; *p* < 0.05; η^2^ = 0.138] with a medium effect size. *Post hoc*-analyses revealed that the HC group significantly differed in attentional orienting to sad faces from the DC group (*p* = 0.036), while the CP lied in between. When looking at single participant groups, the tendency to orient attention first to sad faces was significant in the HC group [*t*(15) = 4.068; *p* = 0.001], however, both other groups showed no significant preference for sad faces in attentional orienting (*p* > 0.05).

### Attentional Maintenance on Emotional Stimuli

**Figure 4:**
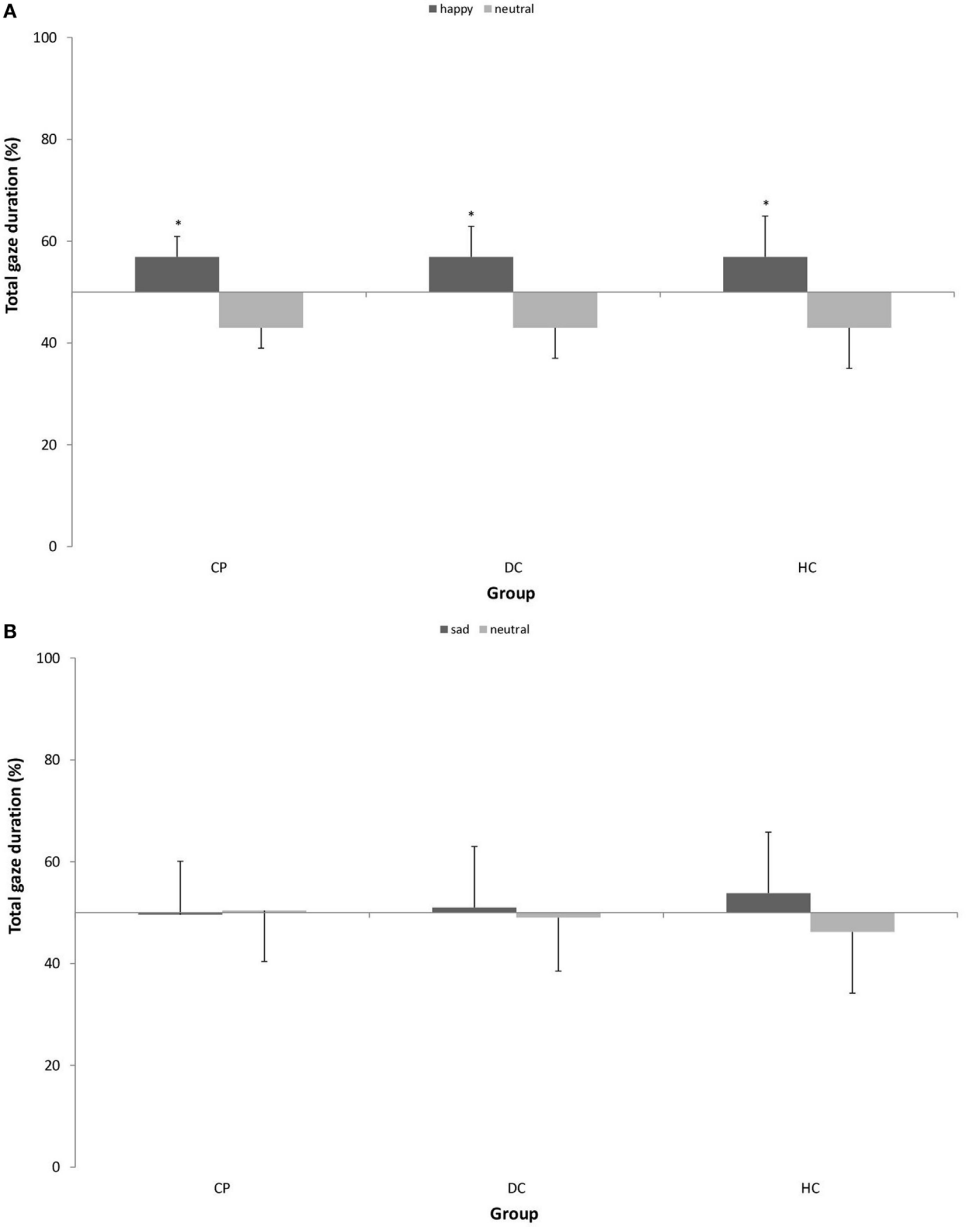
Mean (±SD) percentage of total gaze duration on **(A)** happy vs. neutral facial expression in *n* = 15 patients with chronic pain disorder (CP), *n* = 15 individuals with depressive symptoms (DC), and *n* = 15 healthy individuals (HC) and **(B)** sad vs. neutral facial expression in *n* = 16 CP, *n* = 16 individuals with depressive symptoms (DC), and *n* = 16 healthy individuals (HC). All groups showed significantly longer total gaze duration on happy faces as compared to neutral faces. There was no attentional bias for sad faces (*p < 0.05).

Figure 4 shows the pattern of attentional maintenance on emotional stimuli.

With respect to *happy* vs. neutral faces, we found a main effect of stimulus type [*F* (1, 42) = 30.080; *p* < 0.001; η^2^ = 0.417] with a large effect size, which means, there was a general bias in attentional maintenance on happy faces as compared to neutral faces in all three groups. We found no interaction effect.

With respect to *sad* vs. neutral faces, we found neither a main effect of stimulus type nor an interaction effect. This means, the gaze behavior of all groups stayed statistically on the 50% level of equal distribution, and no attentional bias was detectable.

### Correlations between Attentional Biases and Measures of Alexithymia, Depression, and Pain

No significant correlations were found for both attentional bias scores and the TAS26 total score as a measure of alexithymia or the BDI total score as a measure of depression in the total sample. We also analyzed potential relationships between both attentional bias scores and current pain intensity and strongest pain intensity in the last 4 weeks, respectively, for the CP group only and found no significant correlations.

## Discussion

In the present study, we assessed self-reported alexithymia and attentional processing of standardized emotional (happy and sad) vs. neutral facial expressions using eye-tracking technology in participants with chronic pain as compared to healthy individuals and individuals with depressive symptoms.

As hypothesized, the CP group reported more problems recognizing and describing own emotions on the Toronto Alexithymia Scale than healthy participants, while the control group with elevated depression scores also reported elevated TAS-scores. Our results on alexithymia fit with earlier evidence condensed in two recent reviews which show elevated scores on the Toronto Alexithymia Scale in patients with various chronic pain conditions (6) and with depression (35). This again illustrates the overlap between pain and depression (2)—indeed, alexithymia is not unique to chronic pain disorder, it has been reported in other mental, neurological, and somatic disorders (36–38). Nevertheless, problems associated with alexithymia might represent particular and important disease mechanisms in chronic pain as, e.g., the misinterpretation of emotional arousal as signs of disease or the difficulty in expressing own emotions might contribute to the chronification and maintenance of pain (1, 6). Recently, it has been shown that facets of alexithymia are related to alterations in pain processing in healthy participants (39): participants who reported difficulties in describing feelings had higher pain tolerance scores, while higher values on the cognitive alexithymia scale “externally oriented thinking” were related to lower pain impairment and intensity. However, it is important to consider that alexithymia might not only be a risk factor for chronic pain but also be a consequence of pain experience (1).

With respect to attentional orienting, we were largely able to replicate the *emotionality bias* in healthy participants, that is, healthy participants preferentially looked first on the emotional (happy and sad) face as compared to the neutral face and also preferentially maintained their attention on the happy face. However, for the sad faces, no bias in attentional maintenance was found, which means that all participants showed the same attention deployment toward sad faces as toward neutral faces when analyzing the total presentation period. As already noted by other researchers, this facilitated and prioritized processing of emotional information reflects that emotions are of high relevance for social interactions and survival and might also be rewarding.

We found blunted initial reactions to the emotional face, both with happy and sad expressions, in participants with depressive symptoms, which mean that they did not show a preference for the emotional face like seen in HC. The CP group showed an early attentional bias for happy faces, but not for sad faces. This pattern of early attention deployment partially differs from our expectation that we would find facilitated orienting to negative emotions and less orienting to positive emotions in both groups. However, it is important to note that earlier findings were not based on gaze behavior or used different eye-tracking paradigms, i.e., free viewing tasks without a cue (16). In our paradigm, the location of the emotional stimulus was indicated by a cue. What we found in the DC group—and at least for sad faces also in the CP group—can be interpreted in terms of the anhedonia hypothesis (1, 16): when these individuals are given the opportunity to intentionally guide their initial attention to emotional stimuli in a cuing paradigm, they rather avoid them in comparison to healthy individuals whose attentional system preferentially attends to these highly relevant and potentially rewarding stimuli. This avoidance is especially detrimental for happy faces in individuals with depressive symptoms as eventually turning to positive information could improve their mood—attentional avoidance of positive stimuli might in this group represent a maintenance mechanism of depressive symptoms (16). It is interesting that this bias can already be detected in a group of individuals with mild depressive symptoms as in the present study. Of note, patients with chronic pain did show early preferences for happy faces, comparable to healthy participants, indicating that they show an intact *emotionality bias* at least for positive emotional content. This could be considered an important aspect for coping with chronic pain in multiple ways, as attending to positive emotions could improve mood or could distract attention from pain experience, and especially the perception of a positive emotional face could signal social support. However, patients with chronic pain like individuals with depressive symptoms avoided initial orienting to negative emotions, suggesting that they have special difficulties processing negative emotions. Our findings fit well with the hypothesis that one contributing factor to the maintenance of pain might be the suppression of primary negative emotions, such as sadness (1).

The results do not support our hypothesis concerning attentional maintenance where we had expected to see longer maintenance on sad faces and shorter maintenance on happy faces in CP and DC. This seems, at least at first glance, not consistent with previous research in depressive disorder, however, a recent meta-analysis shows increased maintenance on negative stimuli in depressed individuals was predominantly found in longer presentation times >10 s (16), while we presented our stimulus pairs for 3 s. Additionally, it is important to note that our chronic pain group on average was not severely depressed, and thus, also the control group that was matched according to level of depression found in the CP group and hence was not a group qualifying as clinically depressed. Therefore, it is not surprising that we did not fully replicate findings from earlier research in depressive disorder. Tentatively, we can conclude that maintained attention to emotional information seems intact in chronic pain, although it would be important to test if this still holds true for longer stimulus presentation.

We found signs of an anhedonia bias in CP within the valence rating of stimuli where the CP group rated happy faces as less pleasant than both control groups DC and HC. However, it is interesting that this was not transferred to gaze behavior toward happy faces, as we did not find an attentional bias away from happy faces in this group.

### Strengths and Limitations

This is to the best of our knowledge the first study to investigate attentional processing of emotional information in chronic pain using eye tracking. Recently, the importance to use eye-tracking methodology to investigate attentional processes in pain has been emphasized (7, 22). We have used standardized and well-validated stimulus material, depicting facial expressions of happiness or sadness vs. neutral expressions, hence being able to contrast processing of positive vs. negative primary emotions. A further strength of our design is the inclusion of a second control group of individuals who were carefully matched to the chronic pain patients according to their level of depressive symptoms using the BDI. Our sample of chronic pain patients was a clinical group characterized by long-lasting and intensive pain and heterogeneous with respect to pain localization.

The sample of the present study is not large; however, the effect sizes of all main and interaction effects are medium to large, indicating that the study was not underpowered. The cross-sectional design of our study limits the interpretation of results as being cause or consequence of chronic pain. We have not assessed arousal, therefore, we could not control for this variable and hence, it is difficult to distinguish the respective effects of valence and arousal in the obtained results. Moreover, we have not contrasted a positive vs. a negative emotion as a stimulus pair. It has to be considered that the CP sample was characterized by heterogeneity, which is illustrated by the fact that 40% of these patients fulfilled at least one further diagnosis of a mental disorder.

### Conclusion and Perspectives

We found that patients with chronic pain as compared to healthy participants display different strategic attention deployment to emotional vs. neutral stimuli in early processing stages in a cued eye-tracking paradigm. Patients with chronic pain showed facilitated early attention to positive emotional stimuli (i.e., happy facial expressions), but no facilitated early attention to negative emotional stimuli (i.e., sad facial expressions), while we found no group differences in attentional maintenance on emotional stimuli.

This pattern of results was very similar to that of a second control group of individuals who were matched to the chronic pain patients according to level of depressive symptoms. We found few effects that were unique for chronic pain, rather there were many similarities between CP and DC in all measures of emotional processing in our study. Our study suggests that patients with chronic pain predominantly show alterations in early processing of negative emotions, while these difficulties also generalize to positive emotions in individuals with depressive symptoms. This is in line with the clinical large overlap between pain and depression (2) and points to potential common maintenance mechanisms. The blunted initial reaction to sadness could be interpreted in terms of a failure of the attentional system to attend to evolutionary salient emotional stimuli (i.e., an anhedonia bias) (1, 16) or as an attempt to suppress negative emotions that are difficult to identify, express, or regulate (2). However, it is often hard to disentangle sequence and causality of pain and depression (2). Difficulties in emotion processing in patients with chronic pain could be predominantly due to comorbid depressive symptoms, however, nevertheless, they might represent an important factor contributing to chronification and maintenance of pain and should therefore be addressed by targeted interventions.

Larger studies investigating emotion processing in chronic pain disorder are needed to expand our knowledge on mechanisms and potential consequences for treatment. These studies should include paradigms with longer presentation times of stimuli, tasks addressing different processing stages and competencies of the attentional system and could combine emotional stimuli with stimuli expressing pain, similarly as previously done by Liossi et al. (22). Potential interventions targeting emotion processing in chronic pain could comprise attentional bias modification, similarly as piloted with pain-related stimuli (40) or a range of other non-invasive neuromodulatory interventions, e.g., including mindfulness interventions (41) or emotional exposure techniques (1).

## Ethics Statement

The study was approved by the Ethics Committee of the Medical Faculty of Tuebingen University (approval number: 581/2013 BO2). All study participants have given written informed consent.

## Author Contributions

KG, PE, SZ, and FJ substantially contributed to the conception and design of the work. SP and IS substantially contributed to data acquisition. KG analyzed the data. KG and FJ are responsible for interpretation of data for the work. KG drafted the work. SP, IS, PE, SZ, and FJ critically revised the work for important intellectual content. All authors gave final approval of the version to be published and agreed to be accountable for all aspects of the work in ensuring that questions related to the accuracy or integrity of any part of the work are appropriately investigated and resolved.

## Conflict of Interest Statement

The present research was conducted in the absence of any commercial or financial relationships that could be construed as a potential conflict of interest.

## Abbreviations

ANOVA, analysis of variance; AOI, areas of interests; BDI, Beck Depression Inventory; CP, patients with chronic pain disorder; DC, individuals who were matched to CP according to level of depressive symptoms; HC, healthy controls; KDEF, Karolinska Directed Emotional Faces; PHQ-D, Patient Health Questionnaire; SSRI, selective serotonin reuptake inhibitor; TAS26, Toronto Alexithymia Scale 26; VAS, visual analogue scale.
